# The Sodium-Glucose Co-Transporter 2 (SGLT2) Inhibitor Empagliflozin Reverses Hyperglycemia-Induced Monocyte and Endothelial Dysfunction Primarily through Glucose Transport-Independent but Redox-Dependent Mechanisms

**DOI:** 10.3390/jcm12041356

**Published:** 2023-02-08

**Authors:** Dilvin Semo, Julius Obergassel, Marc Dorenkamp, Pia Hemling, Jasmin Strutz, Ursula Hiden, Nicolle Müller, Ulrich Alfons Müller, Sajan Ahmad Zulfikar, Rinesh Godfrey, Johannes Waltenberger

**Affiliations:** 1 Vascular Signalling, Molecular Cardiology, Department of Cardiology I—Coronary and Peripheral Vascular Disease, Heart Failure, University Hospital Münster, 48149 Münster, Germany; 2 Department of Obstetrics and Gynecology, Medical University of Graz, 8036 Graz, Austria; 3 Department of Internal Medicine III, University Hospital Jena, 07743 Jena, Germany; 4 Department of Cardiology, St. Gregorios Hospital, Parumala, Kerala 689626, India; 5 Department of Physiology, Cardiovascular Research Institute Maastricht (CARIM), 6229 ER Maastricht, The Netherlands; 6 Department of Cardiovascular Medicine, Medical Faculty, University of Münster, 48149 Münster, Germany; 7 Hirslanden Klinik Im Park, Cardiovascular Medicine, Diagnostic and Therapeutic Heart Center AG, 8002 Zürich, Switzerland

**Keywords:** diabetes mellitus, empagliflozin, SGLT-2, monocytes, endothelial cells, vascular dysfunction, chemotaxis, reactive oxygen species (ROS), glucose transport, VEGFR-2, VEGFR-1

## Abstract

Purpose: Hyperglycaemia-induced oxidative stress and inflammation contribute to vascular cell dysfunction and subsequent cardiovascular events in T2DM. Selective sodium-glucose co-transporter-2 (SGLT-2) inhibitor empagliflozin significantly improves cardiovascular mortality in T2DM patients (EMPA-REG trial). Since SGLT-2 is known to be expressed on cells other than the kidney cells, we investigated the potential ability of empagliflozin to regulate glucose transport and alleviate hyperglycaemia-induced dysfunction of these cells. Methods: Primary human monocytes were isolated from the peripheral blood of T2DM patients and healthy individuals. Primary human umbilical vein endothelial cells (HUVECs) and primary human coronary artery endothelial cells (HCAECs), and fetoplacental endothelial cells (HPECs) were used as the EC model cells. Cells were exposed to hyperglycaemic conditions in vitro in 40 ng/mL or 100 ng/mL empagliflozin. The expression levels of the relevant molecules were analysed by RT-qPCR and confirmed by FACS. Glucose uptake assays were carried out with a fluorescent derivative of glucose, 2-NBDG. Reactive oxygen species (ROS) accumulation was measured using the H_2_DFFDA method. Monocyte and endothelial cell chemotaxis were measured using modified Boyden chamber assays. Results: Both primary human monocytes and endothelial cells express SGLT-2. Hyperglycaemic conditions did not significantly alter the SGLT-2 levels in monocytes and ECs in vitro or in T2DM conditions. Glucose uptake assays carried out in the presence of GLUT inhibitors revealed that SGLT-2 inhibition very mildly, but not significantly, suppressed glucose uptake by monocytes and endothelial cells. However, we detected the significant suppression of hyperglycaemia-induced ROS accumulation in monocytes and ECs when empagliflozin was used to inhibit SGLT-2 function. Hyperglycaemic monocytes and endothelial cells readily exhibited impaired chemotaxis behaviour. The co-treatment with empagliflozin reversed the PlGF-1 resistance phenotype of hyperglycaemic monocytes. Similarly, the blunted VEGF-A responses of hyperglycaemic ECs were also restored by empagliflozin, which could be attributed to the restoration of the VEGFR-2 receptor levels on the EC surface. The induction of oxidative stress completely recapitulated most of the aberrant phenotypes exhibited by hyperglycaemic monocytes and endothelial cells, and a general antioxidant N-acetyl-L-cysteine (NAC) was able to mimic the effects of empagliflozin. Conclusions: This study provides data indicating the beneficial role of empagliflozin in reversing hyperglycaemia-induced vascular cell dysfunction. Even though both monocytes and endothelial cells express functional SGLT-2, SGLT-2 is not the primary glucose transporter in these cells. Therefore, it seems likely that empagliflozin does not directly prevent hyperglycaemia-mediated enhanced glucotoxicity in these cells by inhibiting glucose uptake. We identified the reduction of oxidative stress by empagliflozin as a primary reason for the improved function of monocytes and endothelial cells in hyperglycaemic conditions. In conclusion, empagliflozin reverses vascular cell dysfunction independent of glucose transport but could partially contribute to its beneficial cardiovascular effects.

## 1. Introduction

Diabetes mellitus-associated hyperglycaemia is a significant risk factor for developing cardiovascular disease (CVD) and associated cardiovascular mortality [1]. Diabetic vascular disease is responsible for a 2–4-fold rise in the development of coronary artery disease (CAD) [2,3]. Oxidative stress plays a vital role in developing complications associated with diabetes by inducing vascular cell dysfunction [4]. There are a variety of pathways through which hyperglycaemia transduces its deleterious effects downstream. The induction of inflammation through the activation of NF-κB pathway is one of the major determinants contributing to vascular complications in diabetes [5]. Prolonged hyperglycaemia-induced advanced glycation end products (AGEs) through the Receptor for Advanced Glycation End-products (RAGE)–NF-κB pathway contribute heavily to inflammation induction in T2DM [6]. RAGE has also been implicated in mediating the dysfunction of both monocytes [7] and endothelial cells [8]. There is significant cross-talk between oxidative stress induction and AGE-RAGE signalling [9,10].

The sodium-glucose co-transporter 2 (SGLT2) is a major glucose transporter accountable for the renal reabsorption of almost 90% of the glucose from the urine [11]. SGLT-2 inhibitor empagliflozin, is used to treat T2DM and heart failure. It is considered a new therapy for cardiovascular diseases as numerous clinical trials have shown favourable outcomes. These include the EMPA-REG OUTCOME (NCT01131676) [1,12], DAPA-HF (NCT03036124) [13] and the EMPORER-Reduced (NCT03057977) [14]. The EMPA-REG OUTCOME trial demonstrated the ability of empagliflozin to reduce cardiovascular events and overall mortality in T2DM patients with higher cardiovascular risk [1,12,15], and this by far outweighs the benefits of other glucose-lowering T2DM medications such as dipeptidyl peptidase 4 inhibitors or glucagon-like peptide-1 analogues.

Several animal studies have reported the pleiotropic effects of empagliflozin, and this drug is known to give protection against high glucose level-independent diseases such as atherosclerosis [16], heart failure [17,18] and myocardial infarction [19]. Therefore, it is highly likely that the cardiovascular benefits of empagliflozin are not solely through the reduction of blood glucose levels. The favourable pleiotropic effects of empagliflozin have already been described and discussed in several studies [20,21]. Empagliflozin is known to interfere with cellular redox status by attenuating ROS generation [22,23]. Previous studies from our laboratory have shown that in the T2DM environment, monocytes [7] and endothelial cells [24] are dysfunctional due to the accumulation of reactive oxygen species (ROS). Both monocytes and endothelial cells carry out vital functions in cardiovascular physiology, and their function is compromised during T2DM conditions [7,24,25,26]. Indeed, the reversal of endothelial dysfunction and imparting vascular protective effects by empagliflozin has been described in both T1DM [27] and T2DM [28]. However, Empagliflozin’s beneficial effects on monocyte function have not been reported so far.

The increased incidence of CAD in T2DM patients has been linked to the impaired arteriogenesis and angiogenesis found in these patient groups [29,30,31]. Lack of proper VEGF responses contributes to endothelial and monocyte dysfunction in T2DM [24,31,32,33]. The inability of T2DM endothelial cells to respond to VEGF-2 activating growth factors leads to impaired VEGFR-2-dependent processes such as proliferation, migration and angiogenesis [24,31]. Abnormalities of angiogenesis induced by T2DM directly contribute in the pathogenesis of diabetes complications [34]. Furthermore, T2DM individuals have reduced coronary collateral formation compared to non-diabetics [35]. The defective arteriogenesis is hypothesised to be due to the dysfunction of “arteriogenic” cells, leading to the disability of these cells to home to the sites of vessel growth. Monocytes from T2DM patients were found to be defective in their migratory potential towards VEGFA and PlGF-1, previously described as “VEGF resistance” [7,26,33]. VEGF resistance is based on the non-specific activation of downstream signalling pathways in vascular cells. Pre-activation results in these cells’ resistance to respond to more specific signals. As the VEGFA or PlGF-1 responses are very specific for endothelial cells and monocytes, resistance to these growth factors results in monocyte and endothelial dysfunction [36,37].

Considering the importance of oxidative stress in contributing to both monocyte and endothelial dysfunction, the proposed role of empagliflozin as a redox modulator and the ability of empagliflozin to improve cardiovascular outcomes, we hypothesised that empagliflozin could circumvent both monocyte and endothelial dysfunction through a glucose transport-dependent or independent mechanism, thereby improving vascular health. Such a possibility was investigated in this study.

## 2. Results

### 2.1. Primary Human Monocytes and Primary Endothelial Cells Express SGLT-2

**Figure 1:**
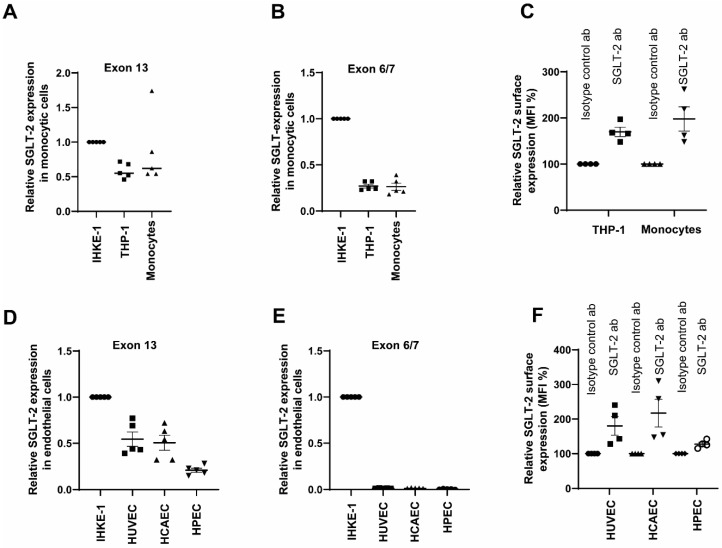
Monocytic and endothelial cells express SGLT-2. (**A**,**B**) CD14++CD16 monocytes isolated from healthy individuals (*n* = 5) and THP-1 monocytic cells (*n* = 5) were analysed for the transcript levels of SGLT-2 using two different primer pairs targeting the exon 13 and exon 6/7 using RT-qPCR. Immortalised Human Kidney Epithelial cells (IHKE1) were used as a positive control. rPLO was used as the house/keeping gene to normalise the gene expression. All data are means ± SEM. (**C**) CD14++CD16 monocytes isolated from healthy individuals (*n* = 4), and THP-1 monocytic cells were analysed by flow cytometry for the surface expression of SGLT-2 compared to the signal from the isotype-specific antibody. The mean fluorescence intensity (MFI) was then quantified. All data are means ± SEM. (**D**,**E**) Human Umbilical Vein Endothelial Cells (HUVEC), Human Coronary Artery Endothelial Cells (HCAEC) and Human fetoplacental Endothelial Cells (HPEC) (*n* = 5 each) were analysed for the transcript levels of SGLT-2 using two different primer pairs targeting the exon 13 and exon 6/7 using RT-qPCR. Immortalised Human Kidney Epithelial cells (IHKE1) were used as a positive control. rPLO was used as the house/keeping gene to normalise the gene expression. All data are means ± SEM. (**F**) Human Umbilical Vein Endothelial Cells (HUVEC), Human Coronary Artery Endothelial Cells (HCAEC) and Human fetoplacental Endothelial Cells (HPEC) (*n* = 5 each) were analysed by flow cytometry for the surface expression of SGLT-2 compared to the signal from the isotype-specific antibody. The mean fluorescence intensity (MFI) was then quantified. All data are means ± SEM.

SGLT-2 is the glucose transporter is primarily expressed in the kidneys on the epithelial cells lining the first segment of the proximal tubule. Since the reports about the expression of SGLT-2 in monocytes and endothelial cells were not robust, we decided to analyse the expression pattern of SGLT-2 in these two cell types. First, we used Immortalised Human Kidney Epithelial cells (IHKE1) as a positive control to detect a positive signal for SGLT-2. We used CD14^++^CD16^−^ primary monocytes and THP-1 monocytic cell line and detected the mRNA levels of SGLT-2 using RT-qCR. We used two sets of primers, one designed to span the boundaries of exons 6 and 7 and another located at exon 13 of SGLT-2, as reported previously [38]. The exon 13 primers were reported robust in amplifying the SGLT-2 gene. Figure 1A,B shows that both primary monocytes and THP-1 monocytic cells express SGLT-2 mRNA. Both set of primers amplified the SGLT-2 gene. Even though only at a 50% expression level compared to the positive control, IHKE-1 cells, both monocytic cells expressed SGLT-2 transcripts. As reported, the exon 13 primer was more efficient in amplifying the SGLT-2 gene. In order to confirm the gene product, we sequenced the product, and it was confirmed to be the SGLT-2 gene (results not shown).

Furthermore, using FACS, we reliably detected the surface levels of the SGLT-2 protein. Similar to the situation in monocytic cells, three different types of endothelial cells, the Human Umbilical Vein Endothelial Cells (HUVEC), Human Coronary Artery Endothelial Cells (HCAEC) and Human fetoplacental Endothelial Cells (HPEC), showed varying degrees of SGLT-2 gene expression, with HPEC expressing the lowest levels (Figure 1C). Interestingly, we detected the SGLT-2 transcript amplification only when we used the exon 13 primers. We could not detect any SGLT-2 transcripts reliably when the exon 6/7 was used (Figure 1D). Nevertheless, using FACS, we detected SGLT-2 protein levels on the surfaces of these three endothelial cell types and the protein expression pattern matched with the transcript levels, with HPEC showing the lowest SGLT-2 expression. (Figure 1E). Taken together, these data confirm that both monocytes and endothelial cells express SGLT-2.

### 2.2. CD14^++^CD16^−^ Monocytes Exposed to Hyperglycemic Conditions Do Not Exhibit an Enhanced Transmigration Phenotype

**Figure 2:**
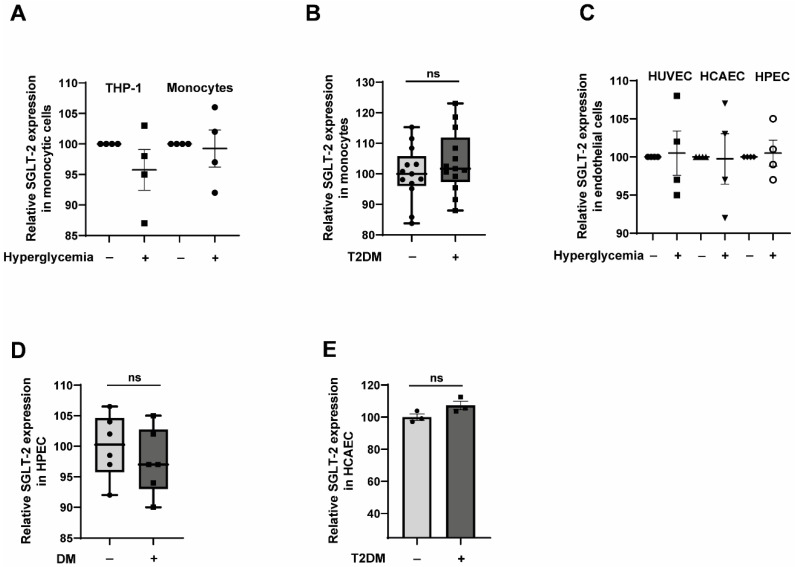
Diabetic conditions do not alter the expression of SGLT-2 in monocytes and endothelial cells. (**A**) CD14^++^CD16^−^ monocytes isolated from healthy individuals (*n* = 4) and THP-1 monocytic cells (*n* = 4) were exposed to either normoglycemic or hyperglycaemic conditions for 48 h. The cells were then analysed for the transcript levels of SGLT-2 using primer pairs targeting the exon 13 using RT-qPCR. rPLO was used as the house/keeping gene to normalise the gene expression. All data are means ± SEM. (**B**) CD14++CD16 monocytes isolated from T2DM patients (*n* = 12) and non-T2DM individuals (*n* = 12) were analysed for the transcript levels of SGLT-2 using primer pairs targeting the exon 13 using RT-qPCR. rPLO was used as the house/keeping gene to normalise the gene expression. (**C**) Human Umbilical Vein Endothelial Cells (HUVEC), Human Coronary Artery Endothelial Cells (HCAEC) and Human fetoplacental Endothelial Cells (HPEC) (*n* = 4 each) were exposed to either normoglycemic or hyperglycaemic conditions for 48 h. The cells were then analysed for the transcript levels of SGLT-2 using primer pairs targeting the exon 13 using RT-qPCR. rPLO was used as the house/keeping gene to normalise the gene expression. All data are means ± SEM. (**D**) HPECs (Human fetoplacental Endothelial Cells) isolated from gestational diabetes patients (*n* = 6) and non-diabetic individuals (*n* = 6) were analysed for the transcript levels of SGLT-2 using primer pairs targeting the exon 13 using RT-qPCR. rPLO was used as the house/keeping gene to normalise the gene expression. (**E**) HCAECs (Human Coronary Artery Endothelial Cells) isolated from T2DM patients (*n* = 3) and non-T2DM individuals (*n* = 3) were analysed for the transcript levels of SGLT-2 using primer pairs targeting the exon 13 using RT-qPCR. rPLO was used as the house/keeping gene to normalise the gene expression. All data are means ± SEM. ns = non-significant.

Since there are reports that diabetic conditions upregulate the expression of SGLT-2 [39], we wondered whether hyperglycaemic conditions or diabetes could modulate the expression of SGLT-2 in monocytes and endothelial cells. For that, we used monocytic cells and endothelial cells cultured under hyperglycaemic conditions in vitro. In addition, we also used CD14^++^CD16^−^ monocytes isolated from T2DM patients and human fetoplacental endothelial cells (HPEC) isolated from gestational diabetes patients. As shown in Figure 2A, hyperglycaemic conditions did not alter the expression of SGLT-2 transcripts in both THP-1 monocytic cells and primary monocytes. Furthermore, T2DM monocytes did not reveal any significant modulation of SGLT-2 expression (Figure 2B).

Similarly, the in vitro hyperglycaemia treatment of HUVEC, HCAEC and HPEC did not alter the levels of SGLT-2 (Figure 2C). Again, neither gestational DM nor T2DM conditions were found to alter the expression levels of SGLT-2 (Figure 2D,E). These data indicate that diabetic conditions do not alter the SGLT-2 expression levels in monocytes and endothelial cells.

### 2.3. SGLT-2 Is Weakly Involved in the Glucose Transport in Both Monocytes and Endothelial Cells

**Figure 3:**
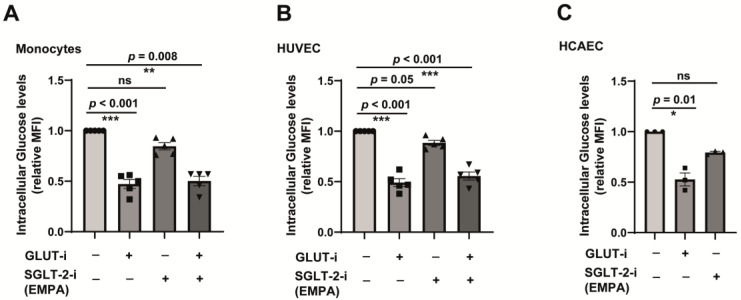
SGLT-2-dependent glucose transport in monocytes and endothelial cells. (**A**) CD14^++^CD16^−^ monocytes were starved without glucose and serum for 2 h, along with either GLUT inhibitor or SGLT-2 inhibitor. Afterwards, the cells were exposed to the fluorescent-tagged derivative of glucose (2-NBDG) for 2–4 h in the presence of GLUT or SGLT-2 inhibitor or both in combination. Cells were then washed and analysed for intracellular fluorescence using FACS or fluorescence plate reader. *n* = 5. All data are means ± SEM. (**B**) HUVECs were starved without glucose and serum for 2 h, along with either GLUT inhibitor or SGLT-2 inhibitor. After that, the cells were exposed to the fluorescent-tagged derivative of glucose (2-NBDG) for 2–4 h in the presence of a GLUT or SGLT-2 inhibitor or both in combination. Cells were then washed and analysed for intracellular fluorescence using FACS or fluorescence plate reader. *n* = 5. All data are means ± SEM. (**C**) HCAECs were starved without glucose and serum for 2 h, along with either GLUT inhibitor or SGLT-2 inhibitor. Afterwards, the cells were exposed to the fluorescent-tagged derivative of glucose (2-NBDG) for 2–4 h in the presence of GLUT or SGLT-2 inhibitor or both in combination. Cells were then washed and analysed for intracellular fluorescence using FACS or fluorescence spectroscopy. *n* = 5. All data are means ± SEM. ns = non-significant. * *p* < 0.05, ** *p* < 0.01 and *** *p* < 0.001.

Since we detected the transcripts and surface expression of SGLT-2 in monocytes and endothelial cells, we wondered about the potential function of these transporters in these cells. In order to understand the role of SGLT-2 as a glucose transporter, we used fluorescent-tagged glucose derivative (2-NBDG) and carried out glucose transport assays. Since GLUT-dependent transport has been reported in monocytes [40] and endothelial cells [41], we employed a GLUT1 inhibitor to understand GLUT-dependent glucose transport. As shown in Figure 3A, inhibition of GLUT resulted in the significant reduction of 2-NBDG accumulation in primary monocytes. However, the inhibition of SGLT-2 using empagliflozin resulted in a steady but not significant reduction in the 2-NBDG uptake. Both GLUT and SGLT-2 inhibitors did not synergistically influence the glucose uptake (Figure 3A). A similar trend was observed for GLUT inhibition in HUVEC, but the inhibition of SGLT-2 resulted in a meagre but significant difference in the 2-NBDG accumulation (Figure 3B). For HCAEC, SGLT-2 inhibition did not result in a significant difference in the glucose transport but showed a clear tendency in that direction (Figure 3C).

### 2.4. Hyperglycemia-Induced Monocyte Dysfunction Is Oxidative Stress-Dependent, and Empagliflozin Alleviates ROS Accumulation and Reverses Monocyte Dysfunction

**Figure 4:**
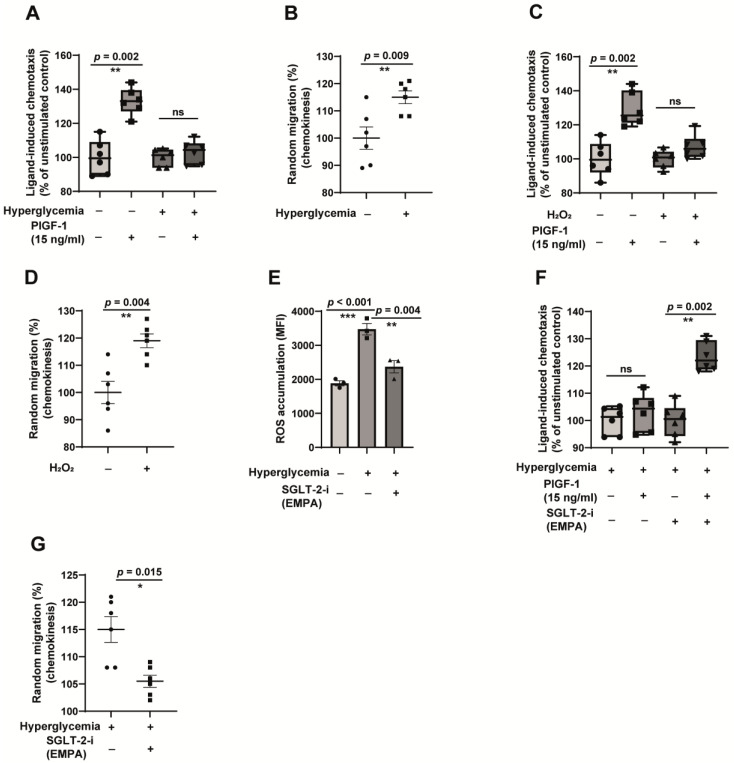
Hyperglycaemia-induced monocyte dysfunction is reversed by empagliflozin through the modulation of oxidative stress. (**A**) CD14^++^CD16^−^ monocytes were cultured in vitro in normoglycemic (5 mM glucose) or hyperglycaemic (30 mM glucose + 100 µM methylglyoxal) for 48 h and were analysed for their ability to undergo chemotaxis (directional migration) towards arteriogenic stimuli PlGF-1. Boyden chamber assays were performed. *n* = 6. (**B**) CD14^++^CD16^−^ monocytes were cultured in vitro in normoglycemic (5 mM glucose) or hyperglycaemic (30 mM glucose + 100 µM methylglyoxal) for 48 h and were analysed for their ability to undergo chemokinesis (random migration). Checkerboard analyses were performed for this. *n* = 6. All data are means ± SEM. (**C**,**D**) CD14^++^CD16^−^ monocytes were cultured in vitro in normoglycemic (5 mM glucose) conditions in the presence of 200 µM H_2_O_2_ for 24 h. After that, the cells were analysed for their ability to undergo chemotaxis towards PlGF-1 and chemokinesis using Boyden chamber assays. *n* = 6. All data are means ± SEM. (**E**) CD14^++^CD16^−^ monocytes were cultured in vitro in normoglycemic (5 mM glucose) or hyperglycaemic (30 mM glucose + 100 µM methylglyoxal) for 48 h. The reactive oxygen species (ROS) accumulated was detected by fluorescence spectroscopy using 5-(and-6)-carboxy-2′,7′-difluorodihydrofluorescein diacetate (H2-DFFDA) reagent. *n* = 3. All data are means ± SEM. (**F**,**G**) CD14^++^CD16^−^ monocytes were cultured in vitro under hyperglycaemic (30 mM glucose + 100 µM methylglyoxal) conditions for 48 h in the presence or absence of 100 ng/mL SGLT-2 inhibitor empagliflozin. After that, the cells were analysed for their ability to undergo chemotaxis towards PlGF-1 and chemokinesis using Boyden chamber assays. *n* = 6. All data are means ± SEM. ns = non-significant. * *p* < 0.05, ** *p* < 0.01 and *** *p* < 0.001.

Monocytes are rendered dysfunctional in T2DM conditions. As shown in Figure 4A, hyperglycaemic monocytes cannot migrate toward a strong arteriogenic stimulus, the placental growth factor-1 (PlGF-1), which is a direct readout indicating monocyte dysfunction. This inability is partly attributed to the ligand-independent activation monocytes resulting in random motility. The random motile monocytes (termed chemokinesis) cannot sense and specifically respond to growth factor stimulation. As expected, the hyperglycaemic monocytes readily undergo chemokinesis (Figure 4B). Oxidative stress was found to be a primary driver of monocyte dysfunction. The exogenous addition of hydrogen peroxide (H_2_O_2_) was found to be sufficient to induce monocytes’ refractoriness to respond to PlGF-1 (Figure 4C). Exactly as in hyperglycaemic conditions, H_2_O_2_ treatment alone was sufficient to induce monocyte chemokinesis (Figure 4D). Since empagliflozin has several pleiotropic effects and is considered to be a redox modulator [28], we hypothesised that empagliflozin could interfere with hyperglycaemia-induced ROS accumulation. As shown in Figure 4E, empagliflozin significantly attenuated hyperglycaemia-induced ROS accumulation. Similarly, hyperglycaemia-induced monocyte dysfunction (Figure 4F) and the induction of monocyte chemokinesis (Figure 4G) were significantly reinstated by empagliflozin. Since empagliflozin does not significantly modulate glucose transport in monocytes, these beneficial effects are independent of the intracellular glucose levels.

### 2.5. Oxidative Stress-Dependent Impairment of VEGFR-2 Contributes to Endothelial Dysfunction in Hyperglycemia, and Empagliflozin Restores VEGFR-2 to Alleviate Endothelial Dysfunction

**Figure 5:**
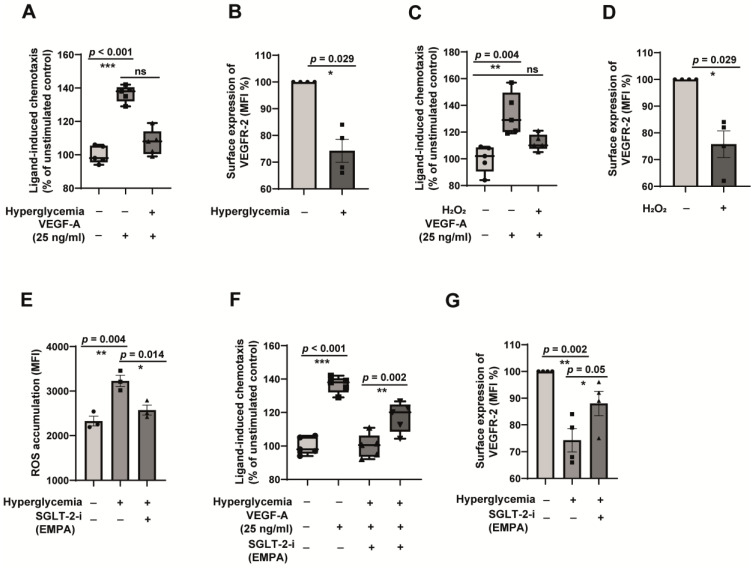
Empagliflozin reverses hyperglycaemia-induced endothelial dysfunction by reducing oxidative stress. (**A**) HUVECs were exposed to in vitro normoglycemic conditions (5 mM glucose) or hyperglycaemic conditions mimicking a diabetic milieu (30 mM glucose + 100 µM methylglyoxal) for 24 h. The cells were then analysed for their ability to undergo chemotaxis (directional migration) towards angiogenic stimuli VEGF-A. Boyden chamber assays were performed. *n* = 5. (**B**) HUVECs were exposed to in vitro normoglycemic conditions (5 mM glucose) or hyperglycaemic conditions mimicking a diabetic milieu (30 mM Glucose + 100 µM methylglyoxal) for 24 h. Thereafter, FACS analysis of the surface expression of VEGFR-2 on hyperglycaemic HUVECs was done. (*n* = 5). All data are means ± SEM. (**C**) HUVECs were exposed to in vitro normoglycemic conditions (5 mM glucose) in the presence of 200 µM H_2_O_2_ for 24 h. After that, the cells were analysed for their ability to undergo chemotaxis towards VEGF-A using Boyden chamber assays. *n* = 5. (**D**) HUVECs were exposed to in vitro normoglycemic conditions (5 mM glucose) in the presence of 200 µM H_2_O_2_ for 24 h. After that, the cells were analysed for the surface expression of VEGFR-2 using FACS. *n* = 4. Data are means ± SEM. (**E**) HUVECs were exposed to in vitro normoglycemic conditions (5 mM glucose) or hyperglycaemic conditions mimicking a diabetic milieu (30 mM Glucose + 100 µM methylglyoxal for 24 h. The reactive oxygen species (ROS) accumulated was detected by fluorescence spectroscopy using 5-(and-6)-carboxy-2′,7′-difluorodihydrofluorescein diacetate (H2-DFFDA) reagent. *n* = 3. All data are means ± SEM. (**F**) HUVECs were exposed to in vitro normoglycemic conditions (5 mM glucose) or hyperglycaemic conditions mimicking a diabetic milieu (30 mM Glucose + 100 µM methylglyoxal) for 24 h in the presence or absence of 100 ng/mL SGLT-2 inhibitor empagliflozin. After that, the cells were analysed for their ability to undergo chemotaxis towards VEGF-A using Boyden chamber assays. *n* = 5 (**G**) FACS analysis of the surface expression of VEGFR-2 of the cells grown under conditions as described for F. *n* = 4. All data are means ± SEM. ns = non-significant. * *p* < 0.05, ** *p* < 0.01 and *** *p* < 0.001.

Similar to dysfunctional monocytes, diabetes conditions render endothelial cells unable to carry out their primary physiological functions. Most of the signals from the physiological functions of the endothelial cells are transduced through the Vascular Endothelial Growth Factor Receptor-2 (VEGFR-2), and defective VEGFR-2 signalling characterises endothelial dysfunction. We tested this. The ability to respond to VEGFR-2 ligand VEGF-A was significantly reduced in hyperglycaemic endothelial cells (Figure 5A). This defect was found to be due to the reduction of VEGFR-2 surface expression (Figure 5B). Indeed, oxidative stress is vital in mediating the refractoriness of endothelial cells to VEGF-A. The treatment of endothelial cells with exogenous H_2_O_2_ readily recapitulated the impaired ability to respond to VEGF-A stimulation (Figure 5C), and the induction of oxidative stress was sufficient to impair the surface expression of VEGFR-2 levels (Figure 5D). Confirming the role of empagliflozin as a redox modulator, hyperglycaemia-induced ROS accumulated was reduced in empagliflozin-treated endothelial cells (Figure 5E). Furthermore, empagliflozin restored the ability of hyperglycaemic endothelial cells to respond to VEGF-A (Figure 5F), and this was due to the improved surface expression of the VEGFR-2 receptor (Figure 5G). Taken together, these data indicate that empagliflozin modulates redox homeostasis in endothelial cells.

### 2.6. A General Antioxidant Improves Cell Function, Whereas Induction of Oxidative Stress Reverses the Beneficial Effects of Empagliflozin

**Figure 6:**
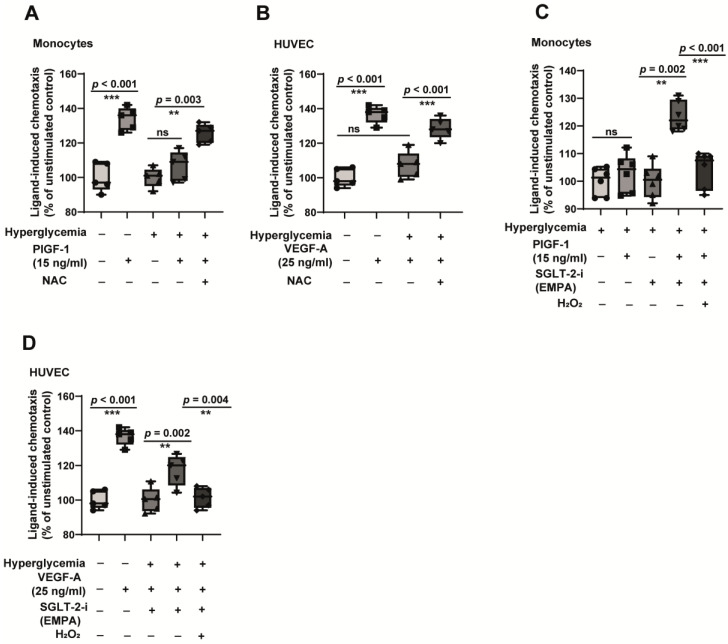
Manipulating the redox status of the cells recapitulates the dysfunction phenotype. (**A**) CD14^++^CD16^−^ monocytes were cultured in vitro in normoglycemic (5 mM glucose) or hyperglycaemic (30 mM glucose + 100 µM methylglyoxal) for 48 h in the presence or absence of 5 mM N-acetylcysteine (NAC) and were analysed for their ability to undergo chemotaxis (directional migration) towards arteriogenic stimuli PlGF-1. Boyden chamber assays were performed. *n* = 5. (**B**) HUVECs were exposed to in vitro normoglycemic conditions (5 mM glucose) or hyperglycaemic conditions mimicking a diabetic milieu (30 mM Glucose + 100 µM methylglyoxal) for 24 h in the presence or absence of 5 mM N-acetylcysteine (NAC). The cells were then analysed for their ability to undergo chemotaxis (directional migration) towards angiogenic stimuli VEGF-A. Boyden chamber assays were performed. *n* = 5. (**C**) CD14^++^CD16^−^ monocytes were cultured in vitro under hyperglycaemic (30 mM glucose + 100 µM methylglyoxal) conditions for 48 h in the presence or absence of 100 ng/mL SGLT-2 inhibitor empagliflozin. In addition, cells were treated with or without 200 µM H_2_O_2_. After that, the cells were analysed for their ability to undergo chemotaxis towards PlGF-1 in the presence of 200 µM H_2_O_2_. *n* = 5. (**D**) HUVECs were exposed to in vitro normoglycemic conditions (5 mM glucose) or hyperglycaemic conditions mimicking a diabetic milieu (30 mM Glucose + 100 µM methylglyoxal) for 24 h in the presence or absence of 100 ng/mL SGLT-2 inhibitor empagliflozin. In addition, cells were treated with or without 200 µM H_2_O_2_. After that, the cells were analysed for their ability to undergo chemotaxis towards VEGF-A using Boyden chamber assays in the presence of 200 µM H_2_O_2_. *n* = 5. ns = non-significant. ** *p* < 0.01 and *** *p* < 0.001.

From the results described so far, it seems likely that the modulation of oxidative stress by empagliflozin contributes to improving cell function in hyperglycaemic conditions. We used a very commonly used antioxidant, N-acetyl cysteine (NAC), in our system. In fact, NAC is known to impart a protective effect against diabetes-associated cardiovascular complications [42]. As hypothesised, the application of NAC reversed hyperglycaemia-induced monocyte and endothelial dysfunction (Figure 6A,B). NAC, such as empagliflozin, significantly restored both the impairment of PlGF-1 and VEGF-A responses. This indicated that oxidative alleviation is central to improving vascular cell function. Next, we asked if we were able to suppress the beneficial effects of empagliflozin by the induction of oxidative stress. For that, we used H_2_O_2_. Oxidative stress significantly attenuated the improvement of monocyte and endothelial cell function induced by empagliflozin in hyperglycaemic conditions (Figure 6C,D). These data further confirmed the role of empagliflozin as a redox regulator in monocytes and endothelial cells.

## 3. Discussion

The present study demonstrates that the two important cell types, the monocytes and endothelial cells, are dysfunctional in hyperglycaemic conditions. These cells are required for vascular repair processes and contribute to atherosclerosis development in diabetic patients when dysfunctional. Empagliflozin, the SGLT-2 inhibitor—developed to attenuate the glucose reabsorption by the kidneys as a strategy to reduce blood glucose levels in diabetes patients—improves both hyperglycaemia-induced monocyte and endothelial cell dysfunction by glucose transport-independent mechanisms. Even though monocytes and endothelial cells express functional SGLT-2, glucose transport is not primarily mediated through SGLT-2 but via GLUT. Empagliflozin interferes with the hyperglycaemia-induced oxidative stress induction and attenuates ROS accumulation. Empagliflozin-treated hyperglycaemic monocytes displayed attenuated chemokinesis and were readily responding to arteriogenic stimuli.

Similarly, empagliflozin-treated endothelial cells displayed improved VEGFR-2 receptor levels on the cell surface and responded robustly to angiogenic stimuli. The attenuation of arteriogenesis and angiogenesis is a hallmark of diabetes mellitus contributing to micro- and macrovascular complications [30,34]. Other than interfering with the glucose transport in the proximal kidney tubules and contributing positively to the reduction of glucotoxicity-dependent alterations in cell function, the results described here highlight the pleiotropic effects of empagliflozin and can be a contributing pathway through which this drug offers protection in diabetic and heart failure patients.

Even though SGLT-2 expression is primarily confined to the kidneys, its expression has been reported in various other cell types, including endothelial cells [23,43] and smooth muscle cells [44]. However, the expression of SGLT-2 on monocytes and monocytic cell lines was not reported. Here, we detected the mRNA and protein expression of SGLT-2 in both primary monocytes and several primary endothelial cell types. These data confirm that SGLT-2 is expressed in vascular cells. SGLT-2 expression is regulated dynamically, and several biochemical stimuli such as TNFα and Ang-II have been reported to increase its expression [23,45]. Furthermore, there are reports about the upregulation of SGLT-2 in diabetes [46,47] and heart failure patients [48]. However, our investigations using in vitro hyperglycaemic conditions and monocytes and endothelial cells from T2DM patients did not reveal any differences in the expression pattern of SGLT-2, indicating that diabetes conditions do not stimulate SGLT-2 expression in these cells. The signalling pathways responsible for the induction of SGLT2 in monocytes and endothelial cells have not been delineated.

We identified that the SGLT-2 expressed on monocytes and endothelial cells are also functionally active. Even though to a very low level, the inhibition of SGLT-2 using empagliflozin resulted in slightly altered glucose transport in both these cell types. Although the differences were minimal, there was always a tendency to downgrade glucose transport. This indicates that SGLT-2 is able to transport glucose in these cells, albeit to a lower level. These also validate our data that the positive effect of empagliflozin on monocyte and endothelial function is not secondary to the reduction of glucose transport. As expected, GLUT inhibition significantly blocked glucose transport in both monocytes and endothelial cells. This is in line with the published data on the vital role of GLUT in transporting glucose [49,50].

This study’s most exciting and novel finding is the influence of empagliflozin in improving monocyte function in hyperglycaemic conditions. Even though several animal studies have shown that empagliflozin is able to improve endothelial function [27,28,51], its positive effects on monocyte function have not yet been reported. Furthermore, most of the positive effects of empagliflozin on endothelial function in vivo are also secondary to its role in reducing glucotoxicity. However, we report that empagliflozin imparts positive effects on endothelial cells and monocytes through a pleiotropic mechanism. Indeed, this ambiguous role of empagliflozin has been reported in several studies [20]. Our data refer to the specific contribution of empagliflozin in alleviating the oxidative stress-dependent induction of monocyte and endothelial cell function. Further studies are required to evaluate the improvement of endothelial function in vivo using the flow-mediated dilation (FMD) method [52].

Translational data suggest that SGLT2 inhibitors may positively affect plaque composition and burden through the reduction of inflammatory and cell adhesion pathways. Still, human data are not available to make solid conclusions. However, endothelial dysfunction represents an important mechanism underlying heart failure with preserved ejection fraction (HFpEF) [53]. Impaired coronary microvascular function is strongly associated with the severity of heart failure [54]. Even though published data do not completely support a causative role for monocytes and heart failure, their role in atrial fibrillation (AF) has been postulated [55]. Therefore, the improvement of both endothelial and monocyte function by empagliflozin could improve heart failure outcomes.

The modulation of oxidative stress by empagliflozin is a pleiotropic mechanism on which several positive effects of this drug could be based. Such a possibility is currently being tested in patients with type 2 diabetes (EMPOX study) in a clinical trial. (ClinicalTrials.gov Identifier: NCT02890745). Our data confirm the notion that empagliflozin can reduce the ROS accumulation induced by hyperglycaemia. This could be secondary to reducing oxidative stress-inducing machinery such as NADPH oxidases (NOXs) or improving the antioxidant system [56]. The dysfunction of monocytes and endothelial cells induced by hyperglycaemia is oxidative stress-dependent [24,33], and the complete alleviation of this dysfunction phenotype of empagliflozin demonstrates its function as a redox regulator. The alleviation of the beneficial effects of empagliflozin by the induction of oxidative stress demonstrates that the beneficial effects of empagliflozin are redox-dependent. Since functionally active monocytes and endothelial cells could carry out a wide array of repair and regeneration processes in diabetes, empagliflozin-mediated improvement of these two cell types would contribute to the beneficial aspects of empagliflozin. Further investigations are necessary to understand how empagliflozin is able to impart its effects as a redox modulator.

## 4. Materials and Methods

### 4.1. Monocyte Isolation from Clinical Cohorts and Healthy Individuals

CD14^++^CD16^−^ human monocytes were isolated from healthy donors and from non-T2DM individuals or T2DM patients according to a published protocol [33] using Magnet-assisted cell sorting (MACS) using negative selection with the human Monocyte Isolation Kit II from Miltenyi Biotec (Bergisch Gladbach, Germany). The study was approved by the scientific and ethics committee of the University of Münster and the University of Jena and conformed to the principles of the Declaration of Helsinki. Written informed consent was obtained from all donors by the blood bank, and thrombocyte reduction filters were provided anonymously without sharing personal and detailed information. The purity of isolated cells was confirmed by FACS, and they were around 98% pure. The clinical characteristics are described in detail in Supplementary Table S1.

### 4.2. Human Umbilical Vein Endothelial Cells Isolation and Ethics

Human umbilical vein endothelial cells (HUVEC) were isolated from anonymously acquired umbilical cords according to the Declaration of Helsinki, “Ethical Principles for Medical Research Involving Human Subjects” (1964), as described previously [57]. The study was approved by the Jena University Hospital Ethics Committee (no. 3130-05/11), and donors were informed and gave written consent. For cell preparation, umbilical cord veins were cleaned with 0.9% NaCl solution, and cells were detached with 0.01% collagenase dissolved in M199 for 3 min at 37 °C. Veins were then rinsed with M199/10% FCS, and the cell suspension was centrifuged (500× *g*, 6 min). The pellet was resuspended in M199/10% FCS and seeded on a cell culture flask coated with 0.2% gelatine. After 24 h, cells were washed and cultured in full growth medium (M199, 20% FCS, 7.5 U/mL heparin, 100 U/mL penicillin and 100 µg/mL streptomycin).

### 4.3. Human Fetoplacental Endothelial Cell Isolation from Clinical Cohorts and Healthy Individuals

Primary HPEC were isolated from arterial vessels of human term placentas obtained from healthy and GDM pregnancies, as described previously [58]. In brief, arterial vessels from the apical surface of the chorionic plate were dissected and cells were isolated by perfusion of the arteries with Hank’s balanced salt solution (HBSS, Invitrogen, Waltham, MA, USA) containing 0.1 U/mL collagenase, 0.8 U/mL dispase II (Roche, Basel, Switzerland) and 10 mg/mL penicillin/streptomycin for 8 min. Digested suspension was centrifuged (200× *g*, 5 min), the cell pellet resuspended in EBM-2 Media supplemented with the EGM-2 MV Bullet Kit (Lonza, Basel, Switzerland), containing 5% human heat-inactivated serum of pregnant women instead of FCS and plated on 1% gelatine-coated wells of a 12-well plate. Cells were split into a 12 cm^2^ flask, 25 cm^2^ flask and, finally, 75 cm^2^ flask accordingly when cells were confluent. The identity and purity of HPEC were confirmed by immunocytochemistry staining of specific endothelial markers for von Willebrand factor and CD31 (PECAM1), fibroblast markers (CD90 and TE-7) and smooth muscle cell markers (SMA and Desmin). For maintaining a culture, primary cells were grown in EBM-2 Media supplemented with the EGM-2 MV Bullet Kit containing 5% FCS, and cells split for less than 10 passages were used for experiments. The ethics committee of the Medical University of Graz approved this study (27-265 ex 14/15). All individuals gave voluntary informed consent and underwent an oral glucose tolerance test (OGTT) at 24 weeks of gestation. Control subjects were selected based on negative OGGT. Women with GDM diagnosed according to the WHO/IADPSG criteria, but without other pregnancy complications, were recruited before delivery. All subjects included in the GDM group were managed by diet and lifestyle modifications during the remaining time of pregnancy. The study conforms to the Declaration of Helsinki. Clinical characteristics are listed in Supplementary Table S2.

### 4.4. Monocyte, HUVEC, HCAEC and HPEC Culture

Primary human monocytes were maintained in RPMI-1640 medium (+l-glutamine, d(+)-glucose; Thermo Scientific, Waltham, MA, USA) supplemented with 5 mM glucose, 10% foetal bovine serum (FBS) and 1% penicillin/streptomycin (P/S). For migration experiments, cells were starved for 2–4 h in FBS-free RPMI-1640 medium. Monocytes were kept in an incubator at 37 °C and 5% CO_2_. Normoglycemic medium contained 5 mM glucose and 25 mM mannitol. In hyperglycaemic medium, 30 mM glucose and 100 µM methylglyoxal were used, and the cells were treated for 48 h. HUVECs were cultured in full growth medium (M199, 20% FCS, 7.5 U/mL heparin and 1% penicillin/streptomycin). In general, HUVEC from 2–5 passages were used for the experiments. For migration experiments, cells were starved for 2–4 h in FBS-free M199 medium. Monocytes were kept in an incubator at 37 °C and 10% CO_2_. Normoglycemic medium contained 5 mM glucose and 25 mM mannitol. In hyperglycaemic medium, 30 mM glucose and 100 µM methylglyoxal were used, and the cells were treated for 24 h. HCAECs were obtained from Lonza and were maintained in EBMTM-2 Basal Medium (CC-3156) and EGMTM-2 MV Microvascular Endothelial Cell Growth Medium SingleQuots^TM^ supplements (CC-4147), as per the recommendations of the manufacturer. Cells up to a passage of 6 were used for the experiments. HPECs were grown in EBM-2 media supplemented with the EGM-2 MV Bullet Kit containing 5% FCS, and cells split for less than 10 passages were used for experiments.

### 4.5. Reagents

Cell culture media RPMI 1640 Medium GlutaMAX^TM^ was obtained from Life Technologies. Human VEGF-A and PlGF-1 were from Peprotech. H_2_DFFDA and CellROX were from Life Technologies. Hydrogen peroxide, methylglyoxal, NAC and 2-NBDG were from Sigma Aldrich. All the primers for qPCR were custom synthesised from Sigma Aldrich. Primer sequences are described in Supplementary Table S3.

### 4.6. RNA Isolation and qPCR

For the extraction of RNA, roughly 5–8 × 10^6^ monocytes were used. For in vitro experiments, the RNA was extracted between 8 and 12 h post-cell treatment. Total RNA purification was performed using a NucleoSpin RNA isolation kit (Macherey-Nagel, Dueren, Germany), and cDNA was synthesised using a RevertAid First Strand cDNA Synthesis Kit (Thermo Scientific, Waltham, MA, USA). qPCR was carried out using iTaq™ Universal SYBR^®®^ Green supermix (Bio-Rad, Hercules, CA, USA) in the Connect Real-Time PCR Detection System (Bio-Rad, Hercules, CA, USA). The threshold cycle (Cq) value of each sample was calculated, and the expression of the target gene mRNA relative to rplO was determined by the 2^−ΔΔCt^ method. The sequences of the primers used can be found in Supplementary Table S3.

### 4.7. Glucose Uptake Assay

Cells were equilibrated in glucose-free medium with 1% serum for three hours prior to being treated with 10 µM of 2-NBDG for 30 min at 37 °C, together with GLUT inhibitor or empagliflozin. Cells were washed two times, and the flow cytometry analysis was carried out using Guava easyCyte (Millipore, Burlington, MA, USA).

### 4.8. Monocyte Chemotaxis and Chemokinesis Assay

Chemotaxis assays were performed as described previously [7,59] using a 48-well Boyden chamber (Neuroprobe, Gaithersburg, MD, USA) and Nucleopore PET membrane (Whatman, Maidstone, UK) with 5 µm diameter pores. Cells in a concentration from 0.5 × 10^6^ cells/mL were allowed to migrate for 90 min at 37 °C and 5% CO_2_. The cells that migrated through the pores were counted. For quantification, migrated cells were counted by 20 high-power fields in four wells using the Axioskop 2 Plus microscope (Carl Zeiss, Jena, Germany).

### 4.9. Chemotaxis Assay of Endothelial Cells

For the detection of endothelial cell chemotaxis, a modified 48-well Boyden chamber (Nucleopore) and a polycarbonate membrane with a pore diameter of 8 mm (Nucleopore) were used as described earlier [24,33]. Endothelial cells were cultured for 24 h under normal and high glucose conditions with or without empagliflozin. For the assay, cells were starved in a serum-free M199 medium for 1 h, trypsinised, washed and resuspended in serum-free medium. Cells were seeded in a concentration of 0.35 × 10^6^ cells/mL and allowed to migrate for 1.5 h at 37 °C and 5% CO_2_ with and without 25 ng/mL VEGF-A stimulation_._ Migrated cells were fixed with 99% ethanol for 10 min and stained with Giemsa staining solution. Cells at the upper side of the filter membrane were scraped off. Migrated cells were counted using ZEISS Axioskop 2 Plus at 10X magnification from 3 different wells.

### 4.10. Intracellular Reactive Oxygen Species Detection

HUVECs were seeded in a 12-well plate in different glucose conditions with or without 100 ng/mL empagliflozin for 24–48 h. The method was based on the modification of the published protocol [60]. After that, cells were washed twice with Krebs-Ringer phosphate glucose buffer (KRPG; 145 mM NaCl, 5.7 mM KH_2_PO_4_, 4.86 mM KCl, 0.54 mM CaCl_2_, 1.22 mM MgSO4 and 5.5 mM glucose) and then resuspended in 1 mL KRPG. Carboxy-H_2_DFFDA (20 μM) or CellROX (5 µM) was added; the suspension mixed well and then incubated in dark for 20 to 30 min at room temperature. The subsequent steps were strictly carried out in the dark. The cells were washed twice with KRPG. The fluorescence intensity was quantified with a fluorescence multimode microplate reader (Vector, Perkin Elmer) with excitation at 485 nm and emission at 530 nm or by Guava easyCyte FACS using the FITC-channel (Millipore, Burlington, MA, USA).

### 4.11. Detection of the Surface Expression of VEGFR-2

HUVECs were cultured in normoglycemic or hyperglycaemic conditions with or without 100 ng/mL empagliflozin for 24 h in a 12-well plate. For the assay, the cells were trysinised, washed once with 1X PBS and resuspended in 500 µL of PBS/BSA (0.5% BSA) solution. Fc-R blocking reagent was added for 10 min at room temperature, and 2 µL of PE-VEGFR2 antibody (Miltenyi Biotec, Bergisch Gladbach, Germany) were added and incubated for 15 min at room temperature in the dark, and the FACS analysis was done using Guava easyCyte (Millipore, Burlington, MA, USA).

### 4.12. Statistical Analysis

To analyse the significance of differences in experiments with monocytes isolated from diabetic or healthy individuals/mice, the Mann–Whitney Rank Sum Test (for intergroup comparisons) or Kruskal–Wallis One-Way Analysis of Variance on Ranks with Tukey’s or Dunn’s post hoc correction was used. For all the other experiments, two-sample independent *t*-tests or when multiple comparisons were made, Kruskal–Wallis One-Way Analysis of Variance on Ranks with Tukey’s or Dunn’s post hoc correction was performed. SigmaPlot 12 software was used for the statistical analysis. The level of significance was defined as *p* < 0.05. All other statistics and graphs were generated using GraphPad Prism 8 software.

## 5. Conclusions

In conclusion, using the cell culture model of in vitro hyperglycaemia and T2DM monocytes and T2DM endothelial cells and endothelial cells from gestational diabetes patients, we identified that both monocytes and endothelial cells express SGLT-2 transcripts and harbour functionally active SGLT-2 on their surface. SGLT-2 was not responsible for the glucose transport in these cells. However, the empagliflozin treatment significantly reversed the monocyte and endothelial cell dysfunction induced by hyperglycaemia. This was completely independent of glucose transport but through the reduction of oxidative stress. Mechanistically, empagliflozin attenuated the oxidative stress-dependent chemokinesis of monocytes and restored the surface levels of VEGFR-2 on endothelial cells. Furthermore, our results highlight the pleiotropic role of empagliflozin as a redox modulator.

## Data Availability

Not applicable.

## Supplementary Materials

The following supporting information can be downloaded at: https://www.mdpi.com/article/10.3390/jcm12041356/s1: Table S1. Clinical characteristics of the non-T2DM and T2DM individuals used in the study. Table S2. Clinical characteristics of non-diabetic and diabetic individuals used for the HPEC isolation. Table S3. RT-qPCR primer sequences used in the study.
