# Supplementary material for: The Sodium-Glucose Co-Transporter 2 (SGLT2) Inhibitor Empagliflozin Reverses Hyperglycemia-Induced Monocyte and Endothelial Dysfunction Primarily through Glucose Transport-Independent but Redox-Dependent Mechanisms
Source: J Clin Med. 2023 Feb 8;12(4):1356. doi: 10.3390/jcm12041356 (PMC9962711; doi:10.3390/jcm12041356)
Supplement: Supplementary file 1 [file jcm-12-01356-s001.zip › jcm-2200614-supplementary.pdf]

Supplementary Material

1 Supplementary Figures and Tables

1.1 Supplementary Tables

Supplementary Table S1:

Clinical characteristics of the non-T2DM and T2DM individuals used in the study.

	Non-DM	T2 DM	significance
N	12	12	
Age (years)	56±17.8	63±7.2	n.s.
Sex (male/female)	9/3	8/4	n.s.
BMI (kg/m^2)	26.5±3.2	36.9±4.9	<0.001
HbA1c (%)	5.33±0.25	8.64±1.02	<0.001
Glucose (mmol/L)	5.06±0.44	12.7±4.75	<0.001
Smoking (yes/no)	1/11	1/11	n.s.
Hypercholesterolaemia (yes/no)	6/6	0/12	0.006

Supplementary Table S2:

Clinical characteristics of non-diabetic and diabetic individuals used for the HPEC isolation.

	**Controls**	**Gestational Diabetes Mellitus (GDM)**
**Number of cases**	7	7
**Sex (males/females)**	3/4	4/3
**Maternal characteristics:**		
**Age (years)**	30.42±2.79	32.2±1.94
**Pre-pregnancy BMI**	22.2±2.56	35±4.18*
**BMI at delivery**	26.8±2.10	36±3.96*
**Neonatal characteristics:**		
**Weight (g)**	3540.85±150.78	3697±150.15
**Length (cm)**	51.85±0.91	52±0.64
**Placental characteristics:**		
**Placental weight (g)**	601.42±37.57	677.5±88.06
**Primary cell characteristics:**		
**Passage number**	6.57±0.36	6.16±0.41

*indicates significance between controls and GDM cases

Supplementary Table S3:

RT-qPCR primer sequences used in the study.

Gene	Forward primer sequence (5' -3')	Reverse primer sequence (5' -3')
**hSGLT-2 exon 6/7**	CCGCATCCGCCTCTACCT	CATGTCCACTGAGATCTTGGT GAA
**hSGLT-2 exon 13**	TTCAGTCTCCGGCATAGCAAG	CATCTCCATGGCACTCTCTGG
**hrplO**	AATCTCCAGGGGCACCATT	CGCTGGCTCCCACTTTGT
